# Patients or prisoners? Time to reconsider the voting rights of mentally disordered offenders

**DOI:** 10.1192/pb.bp.115.050781

**Published:** 2016-08

**Authors:** Gareth Rees, James Reed

**Affiliations:** 1 Birmingham & Solihull Mental Health NHS Foundation Trust

## Abstract

Although the Representation of the People Act 2000 permits most psychiatric in-patients to register on the electoral register, transferred prisoners and those admitted to hospital under hospital orders remain disenfranchised by law. This article clarifies the voting rights of individuals receiving in-patient psychiatric care and contends that the selective disenfranchisement of some mentally disordered offenders is problematic, discriminatory and may breach international human rights law. There are therefore strong arguments for the UK government to address this long-standing inequality before the next general election.

By common law persons of unsound mind are neither permitted to stand nor vote in elections.^1^ However, the Representation of the People Act was amended in 2000 to enfranchise voluntary in-patients and those held under the so-called ‘civil’ provisions of the Mental Health Act 1983 (amended 2007).^2^ This means that the vast majority of patients in general adult settings retain equal voting rights to the general public and can exercise their suffrage in person, by postal vote or by proxy. Despite these changes the Representation of the People Act continues to disenfranchise ‘offenders detained in mental hospitals’, meaning that some of those held under the so-called ‘forensic’ sections of the Mental Health Act are disallowed from voting by law. Determining whether or not a patient detained in a secure hospital is eligible to vote is therefore not straightforward, reflecting the range of circumstances in which assessment/treatment can be enforced by the Mental Health Act (Table 1).

**Table 1:** Sections of the Mental Health Act 1983 (amended 2007) and voting eligibility

Section	Description	Eligible to vote?
2	Admission for assessment	Yes
3	Admission for treatment	Yes
35	Remand to hospital for report on accused's mental condition	Yes
36	Remand of accused person to hospital for treatment	Yes
37	Hospital order	No
38	Interim hospital order	No
45A	Hospital direction	No
47	Removal to hospital of persons serving sentences of imprisonment	No
48	Removal to hospital of other prisoners	Yes

## The voting eligibility of patients in secure hospitals

### Patients detained under ‘civil’ sections of the Mental Health Act (Part II)

Although relatively few in number, some patients held in secure hospitals are detained under the civil sections of the Mental Health Act; these provisions are governed by Part II of the Act and refer primarily to Section 2 (‘Admission for assessment’) and Section 3 (‘Admission for treatment’).^3^ These individuals are not diverted to hospital from the criminal justice system yet are deemed to require secure care by virtue of clinical need (e.g. somebody whose risk of harming others cannot be managed safely within either a general adult psychiatric ward or psychiatric intensive care unit). Since the Representation of the People Act now enfranchises voluntary in-patients and all those detained under civil sections of the Mental Health Act, such patients have equal voting rights to the general public (regardless of whether they are being held in a low-, medium- or high-security unit).

### Patients detained under forensic sections of the Mental Health Act (Part III)

The forensic sections of the Mental Health Act apply to mentally disordered offenders (defined by the Crown Prosecution Service as ‘individuals who have a disability or disorder of the mind and have committed or are suspected of committing a criminal offence’)^3^ and are overseen by Part III of the Mental Health Act. Since the Representation of the People Act continues to disenfranchise detained persons in pursuance of their sentences, the voting rights of mentally disordered offenders are determined by which section(s) of the Mental Health Act have been used to authorise treatment. Unsentenced prisoners (i.e. persons awaiting sentencing or immigration detainees) and those held by remand orders are still permitted to register on the UK electoral roll, whereas transferred prisoners and persons detained under the provisions of a hospital order (whether restricted or not) are disallowed from doing so by law.

## To disenfranchise or not to disenfranchise?

In *Hirst v the United Kingdom (No 2) 74025/01* (2005) the European Court of Human Rights acknowledged that the right to vote is not absolute and that British citizens can be lawfully disenfranchised if the justification for this is proportionate and necessary to maintain a fair and representative democracy.^4^ The UK government therefore maintains that it is justified in rescinding the voting rights of two main groups of individuals: (1) those presumed to be incapable of making independent voting decisions (e.g. persons under the age of 18), and (2) those who forfeit some of their civic rights by breaching the social contract between individuals and the State (e.g. prisoners serving custodial sentences) (Box 1).^5^ To be legitimately prevented from voting, mentally disordered offenders detained under Sections 37, 38, 45A and 47 of the Mental Health Act should therefore conform to at least one of these groups.

## Incapacity to make independent and informed decisions

For centuries people with mental disorders were presumed by common law to be incapable of making certain types of decisions for themselves, for instance owning property, marrying, engaging in business transactions.^1^ It could therefore be argued that the Representation of the People Act is justified in disenfranchising some mentally disordered offenders because they are unable to make capacitous voting decisions. Supporters of this assertion cite data from the 2010 UK General Election which showed that only half of the psychiatric patients enfranchised by the Representation of the People Act actually registered on the electoral roll, and only half of those registered then exercised their right to vote on polling day^7^ (compared with 65.1% of the general public).^8^

**Box 1** British citizens unable to vote in UK general elections

- Those under 18 years of age on polling day
- Members of the House of Lords
- Convicted persons detained in pursuance of their sentences (this includes prisoners serving custodial sentences and some mentally disordered offenders)
- Persons found guilty within the previous 5 years of corrupt or illegal practices in connection with an election

Source: The Electoral Commission.^6^

This justification, however, is unsubstantiated. First, mental capacity is not assessed as part of either the voting process or the decision to detain an individual under the provisions of the Mental Health Act; the supposition that some groups of detained patients inherently lack mental capacity is therefore arbitrary and unprincipled. Indeed, the MacArthur study demonstrated that half of those admitted to hospital with a relapse of schizophrenia and more than three-quarters of those admitted with depression actually retain mental capacity.^9^ Second, uptake rates are unhelpful in this context since enfranchisement is as much about giving individuals the right not to vote as it is about the right to vote. The consequences of impaired mental capacity on voting are therefore impossible to measure, particularly as there are several confounding factors why the psychiatric in-patients already enfranchised by the Representation of the People Act may choose not to vote (e.g. lack of awareness, social exclusion, disillusionment with the political process).^10^ In 2008 the Electoral Commission therefore issued guidance that ‘lack of mental capacity is not a legal incapacity to vote’.^11^ The Representation of the People Act is therefore unjustified in disenfranchising mentally disordered offenders on these grounds. Indeed, if the UK government were to introduce a test of mental capacity into the voting procedure, a significant proportion of the general public may also be disallowed from voting by law.

## Punitive disenfranchisement

The more commonly cited justification for disenfranchising mentally disordered offenders is the tenet that convicted persons in pursuance of their sentences should temporarily lose certain civic rights as a punishment for breaking the so-called ‘social contract’ which exists between individuals and the State.^2^ This notion of imposing a ‘civic death’ on convicted prisoners has been practised in the UK for centuries and was enshrined in law by the Forfeiture Act 1870. Although the lawfulness of punitive disenfranchisement has recently been called into question,^4^ the British government continues to support its use.^12^ This may explain why the Representation of the People Act was amended to enfranchise some mentally disordered offenders (i.e. unsentenced prisoners and those given remand orders), but continues to disenfranchise those who have been issued sentences by a court (i.e. transferred prisoners and those given hospital orders). However, the ongoing disenfranchisement of these patients is problematic for the following reasons discussed below: culpability, purpose and lawfulness.

### Culpability

Mentally disordered offenders who have been transferred to hospital after sentencing (i.e. those detained under Sections 45A, 47 or 47/49 of the Mental Health Act) are obligated to return to prison (to serve the remainder of their tariff) once their treatment in hospital is complete. They are therefore recognised by the State as convicted prisoners and are subject to the Representation of the People Act's disenfranchisement of detained persons in pursuance of their sentences. Applying the same logic to those detained by hospital orders however is problematic.

Hospital orders are imposed by courts where it is agreed (on the recommendation of two doctors) that somebody who has been convicted of a crime should be admitted to hospital in order to receive treatment for a serious mental health problem. Those deemed ‘unfit to plead’ may also be issued with a hospital order after a trial of the facts has been heard in court.^13^ Unlike custodial sentences, the duration of a hospital order is neither fixed nor determined by the type of crime which has been committed; instead, the length of time an individual can be detained is defined by their response to treatment (akin with those detained for treatment under the so-called civil sections of the Mental Health Act). Hospital orders also represent a permanent diversion from the criminal justice system, meaning that individuals detained under Section 37 Mental Health Act have no means of being sent to prison once their psychiatric treatment is complete.^13^ The application of hospital orders therefore calls into question the *mens rea* (‘guilty mind’) of patients detained under Sections 37, 37/41 and 38 of the Mental Health Act and challenges the legitimacy of punitively disenfranchising such individuals.

### Purpose

The Mental Health Act is clear that detaining somebody in hospital should be therapeutic, i.e. ‘in the interests of health, safety or for the protection of others’ and that an individual's civic rights should not be infringed unless there is a legitimate and proportional reason for doing so. Seeing as there is no evidence that disallowing an individual from voting has any meaningful value, the Representation of the People Act's disenfranchisement of some mentally disordered offenders is incompatible with these principles. There is also emerging evidence that keeping psychiatric patients enfranchised during periods of hospital treatment is as an important way of reducing social exclusion and enhanced recovery.^7^

### Lawfulness

In 2005 the Chamber of the European Court of Human Rights was asked to consider the lawfulness of punitive disenfranchisement (in the case of *Hirst*) and ruled unanimously that the UK's blanket ban on prisoner voting contravened Protocol 1 Article 3 of the European Convention on Human Rights (the right not to be subjected to ‘inhuman or degrading treatment or punishment’).^4^ Notwithstanding this judgment, the British government has failed to amend its domestic legislation and continues to endorse punitive disenfranchisement, citing that (a) the right to vote is a privilege and (b) that the temporary disenfranchisement of convicted prisoners serves proportionate and legitimate aims, i.e. to prevent crime, punish offences, enhance civic responsibility and promote respect for the law.^12^ However, given the stark differences between convicted prisoners and those detained under the Mental Health Act it is hard to see how any of these justifications apply to patients receiving treatment in hospital (regardless of their legal status). Indeed, the European Commission for Democracy through Law (also known as the Venice Commission) has stated that excluding a person from voting on the basis of a disability (in this case the presence of a mental disorder) is a form of discrimination which engages Article 29 of the Convention on the Rights of Persons with Disabilities.^14^

Despite mounting pressure on the UK government to widen the franchise, the prime minister stated that he would never be willing to support the enfranchisement of restricted patients (i.e. mentally disordered offenders whose detention is subject to Home Office restrictions), describing them as ‘some of the most dangerous people currently detained’.^12^ This statement is not only unhelpful (given that disallowing ‘dangerous’ individuals from voting in UK general elections neither mollifies these risks nor protects the public from harm), but also serves to reinforce the stigmatisation of those with mental disorders and the assumption that they are in some way less entitled to the same rights as the general public.

Since the publication of the *Hirst* judgment there have also been concerns that permitting mentally disordered offenders to vote may unduly influence the results of elections held in small constituencies which contain large secure hospitals.^1^ This argument is flawed for several reasons. First, in order for minority groups to have a political voice it is important that they are not arbitrarily excluded from the voting process; this is a basic tenet of democracy.^15^ Second, given that large secure hospitals such as Broadmoor Hospital only hold approximately 200 patients, the outcome of most constituency ballots would be unaffected by a change in voting turnout of this magnitude.^16^ Third, there is no empirical evidence to suggest that mentally disordered offenders are more likely to hold unusual or extreme political views compared with the general public.

## Conclusions

Although the UK declared universal suffrage in 1928, the right to vote in general elections is not extended to all British citizens. Whereas amendments to the Representation of the People Act in 2000 enfranchised those admitted to secure hospitals by remand orders and the so-called civil provisions of the Mental Health Act, transferred prisoners and those receiving hospital orders remain disallowed from voting by law. The revocation of these rights is based on the 19th-century notion of punishing convicted criminals with a ‘civic death’ (Forfeiture Act 1870), but more recent case law has established that the blanket disenfranchisement of convicted prisoners contravenes international human rights legislation.^4^

This editorial highlighted several key differences between convicted prisoners and mentally disordered offenders; these differences render the arbitrary disenfranchisement of individuals detained under Sections 37, 38, 45A and 47 Mental Health Act problematic, unhelpful and discriminatory. Although the voting rights of psychiatric patients are sadly unlikely to be high on the political agenda, there is an emerging consensus of opinion that the enfranchisement of psychiatric in-patients supports autonomy, challenges stigmatisation and reduces some of the social exclusion facing individuals diagnosed with mental disorders.^7,10^ If the government is serious about tackling healthcare inequalities and creating a meaningful democracy in the UK, it should amend the Representation of the People Act to enfranchise all mentally disordered offenders, irrespective of the circumstances of their detention.
